# Supplementary material for: Myo-Inositol Reverses TGF-β1-Induced EMT in MCF-10A Non-Tumorigenic Breast Cells
Source: Cancers (Basel). 2023 Apr 15;15(8):2317. doi: 10.3390/cancers15082317 (PMC10136889; doi:10.3390/cancers15082317)
Supplement: Supplementary file 1 [file cancers-15-02317-s001.zip › cancers-2338986-supplementary.pdf]

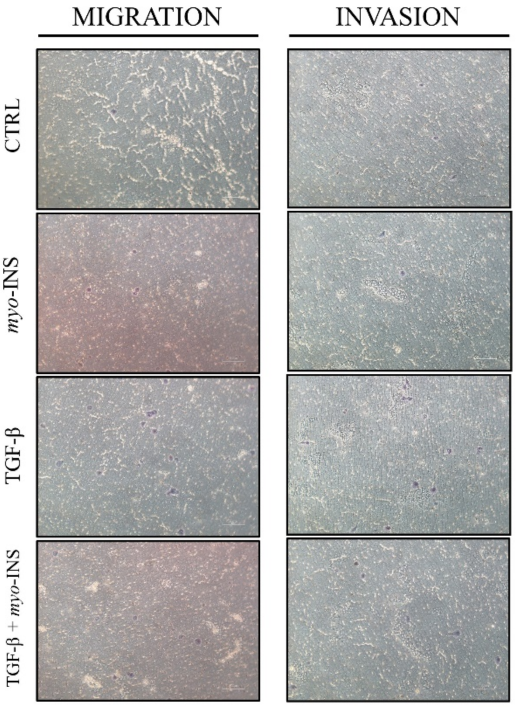

SUPPLEMENTARY

Figure S1. *Myo-inositol inhibits motility and invasiveness upon TGF-β1 induction*. Micrographs are representative of MCF-10A cells migration and invasion transwell assays. Scale bars: 100 µm.

Table S1. List of Primers used

GENE		SEQUENCE
**E-cad**	FW	5' – GAGGGGTTAAGCACAACAGC – 3'
	RV	5' – GCACCTGACCCTTGTACGTG – 3'
**N-cad**	FW	5' – GAGGCTTCTGGTGAAATCGC – 3'
	RV	5' – AGAAGAGGCTGTCCTTCATGC – 3'
**COL1A1**	FW	5' – GCCAAGACGAAGACATCCCA – 3'
	RV	5' – CAACACCTTGCCGTTGTCG – 3'
**FN**	FW	5' – GAGCTATTCCCTGCACCTGA – 3'
	RV	5' – CGTGCAAGGCAACCACACT – 3'
**PI3K**	FW	5' – AGGGTGCTAAAGAGGAACACTG – 3'
	RV	5' – CTGGCCAAAGATTCAAAGCCA – 3'
**SNAI1**	FW	5' – TGGTTCTTCTGCGCTACTGC – 3'
	RV	5' – TTAGGCTTCCGATTGGGGTC – 3'

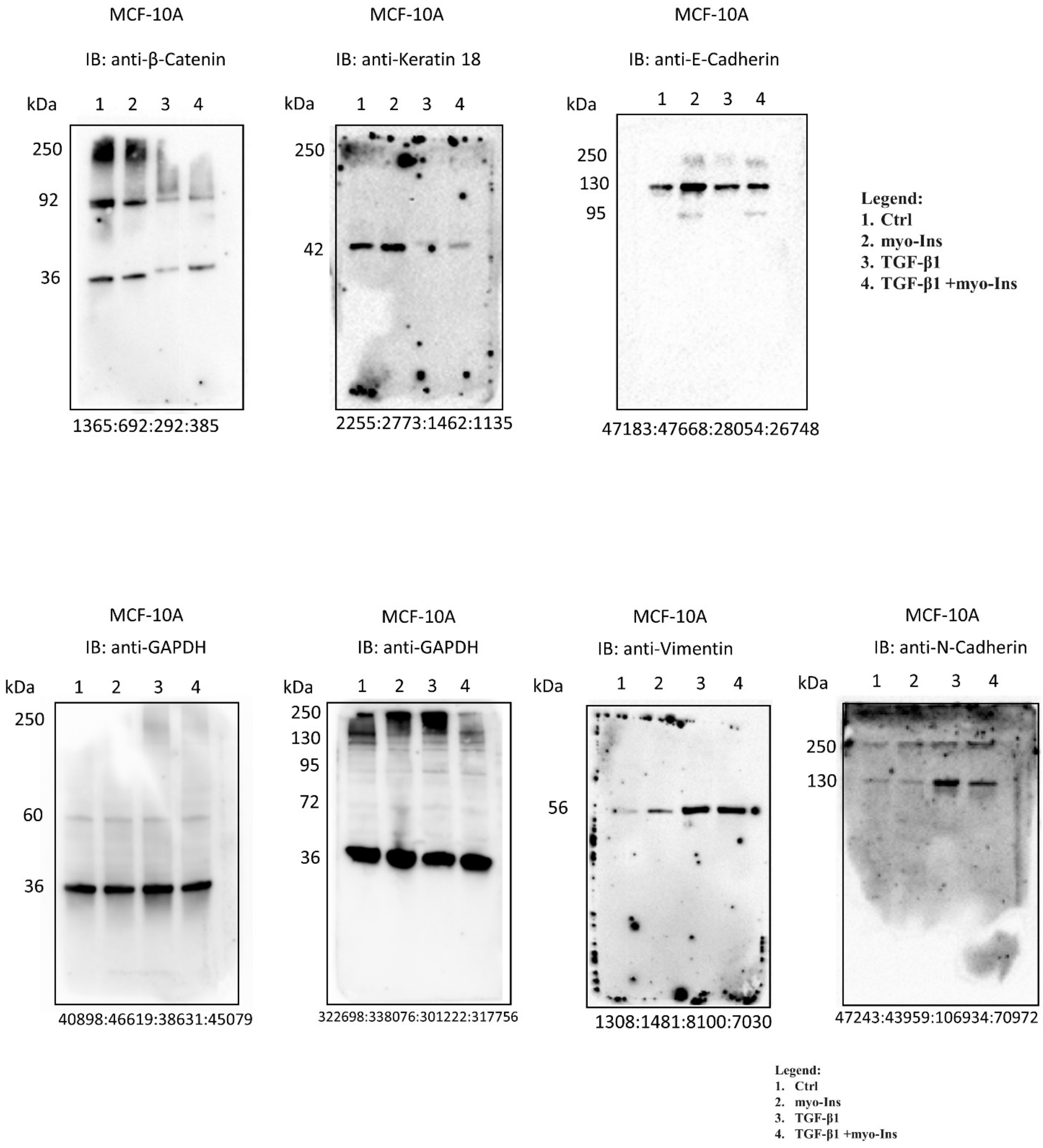

Figure S2. Whole of western blot assays.
